# Pulmonary hypertension in the global population of adolescents and adults living with HIV: a systematic review and meta-analysis

**DOI:** 10.1038/s41598-019-44300-5

**Published:** 2019-05-24

**Authors:** Jean Joel Bigna, Jobert Richie Nansseu, Jean Jacques Noubiap

**Affiliations:** 1 Department of Epidemiology and Public Health, Centre Pasteur of Cameroon, Yaounde, Cameroon; 2 School of Public Health, Faculty of Medicine, University of Paris Sud XI, Le Kremlin Bicêtre, France; 3 Department of Public Health, Faculty of Medicine and Biomedical Sciences, University of Yaounde I, Yaounde, Cameroon; 4 Department of Disease, Epidemics and Pandemics Control, Ministry of Public Health, Yaounde, Cameroon; 5 Department of Medicine, University of Cape Town and Groote Schuur Hospital, Cape Town, South Africa

**Keywords:** Epidemiology, Respiratory tract diseases

## Abstract

A systematic review and meta-analysis was conducted to estimate the prevalence of PH in adolescents and adults living with HIV at the global level. PubMed, EMBASE, Web of Science, and Global Index Medicus were searched to identify articles published until November 4, 2018. PH had to be investigated with transthoracic echography or right heart catheterization (RHC). A random-effects model was used to pool individual studies. Overall, 25 studies with 42,642 participants from 17 countries were included. One study reported the prevalence of PH among HIV-infected adults based on RHC: 0.5% (95% confidence interval (CI): 0.3–0.6). The global prevalence of PH based on echography was 8.3% (95% CI: 4.6–12.8; 22 studies) among HIV-infected adults. In subgroup analysis, there was no difference between regions, human development indicator, and HIV burden in countries. Among HIV-infected adolescents, the prevalence of PH based on echography was 14.0% (95% CI: 2.2–33.1; 2 studies). This study suggests a high prevalence of PH in the global adolescent and adult population infected with HIV. As such, PH in this vulnerable population should be prioritized by HIV healthcare providers, policy makers and stakeholders for improved detection, overall proper management and efficient control.

## Introduction

Pulmonary hypertension (PH) is a hemodynamic condition characterized by the increase in vascular resistance in the pulmonary circulation. Accordingly, a mean pulmonary arterial pressure ≥25 mmHg in right heart catheterisation (RHC) defines this condition^1^. Present estimates suggest a pulmonary hypertension prevalence at about 1% in the global population^2^. PH is classified in five groups based on pathophysiology, clinical presentation, and therapeutic conditions: pulmonary arterial hypertension (group 1), PH due to left-sided heart disease (group 2), PH due to lung disease or hypoxia (group 3), chronic thromboembolic PH and other pulmonary artery obstructions (group 4), and PH with unclear and/or multifactorial mechanisms (group 5)^1^.

To date, it is unclear whether HIV infection is a cause or contributor of PH. Indeed, HIV-associated PH which is part of the group 1 classifications is an important form of lung disease which is usually overlooked in routine clinical practice due to non-specific symptoms especially at early stage^1^. The widespread use of combined antiretroviral treatment has improved the survival of HIV-infected people leading to the emergence of chronic non-communicable diseases such as PH^3^. The exact pathogenesis of HIV-associated PH is still unknown, but HIV proteins such as Tat and Nef are probably implicated. This condition has a poor prognosis, leading progressively to right heart failure and death^4^.

To the very  best of our knowledge, there is no previous study which has attempted to systematically summarize data on the burden of PH in the HIV population. Such knowledge would help healthcare providers, policy makers and stakeholders to improve detection, overall proper management and efficient control of PH in people living with HIV. Therefore, we conducted the present review, which aimed at estimating at the global level the prevalence of PH in adolescents and adults living with HIV.

## Methods

This review was registered in the International Prospective Register of Systematic Reviews (PROSPERO) under the registration number CRD42018115800. We used the Preferred Reporting Items for Systematic Reviews and Meta-analysis (PRISMA) guidelines as template to report this review^5^.

### Search strategy and selection criteria

In this systematic review with meta-analysis, we searched EMBASE, PubMed, Web of Science (Web of Science Core Collection, Current Contents Connect, KCI-Korean Journal Database, SciELO Citation Index, Russian Science Citation Index), and Global Index Medicus to identify studies published until November 4, 2018 with no language restriction. The initial search strategy (Supplementary Table 1) was designed for PubMed and was adapted for other databases. The search strategy was based on the combination of relevant text words and medical subject headings including “HIV”, “AIDS”, “pulmonary hypertension”, and “pulmonary arterial hypertension”. Moreover, references of all relevant articles were scrutinized to identify potential additional data sources.

We included cross-sectional, case-control, and cohort studies reporting the prevalence of PH in adolescents and/or adults living with HIV, or enough information to compute this estimate. We also included studies investigating factors associated with PH in people living with HIV. We considered studies in which PH had been diagnosed with right heart catheterization. We also considered studies in which transthoracic echocardiography had been used as the  screening tool. We excluded reviews, commentaries, editorials, studies lacking key data and/or explicit method description as well as studies in which relevant data on PH in people living with HIV were not available even after contacting the corresponding author at least twice.

Two reviewers (J.J.B. and J.R.N.) independently screened the titles and abstracts of articles for eligibility. Full texts of potentially eligible articles were retrieved and screened for final inclusion. Disagreements between the two reviewers were solved by discussion.

### Data abstraction and analysis

A standardized and pretested data extraction form was used by two reviewers (J.J.B. and J.R.N.) to independently extract data from individual studies. The information which was abstracted from each paper included the last name of the first author, year of publication, country, study design, period of participants’ inclusion, sampling method, timing of data collection, HIV related data, diagnostic method, mean or median age, proportion of males, sample size, number of patients diagnosed with PH and factors associated with PH.

A meta-analysis was used to summarize data concerning the prevalence of PH in HIV. Accordingly, study-specific estimates were pooled through a Der Simonian and Laird random-effects meta-analysis model to obtain an overall summary estimate of the prevalence across studies, after stabilizing the variance of individual studies using the Freeman-Tukey double arc-sine transformation^6^. Prevalence estimates were expressed with their 95% confidence interval and 95% prediction interval. We pooled data from adults and adolescents (<18 years) separately.

Heterogeneity between studies was assessed with Cochran’s Q, *I*^2^, and *H* statistics^7^. The *I*^2^ statistic estimates the percentage of total variation across studies due to true between-study differences rather than chance. Generally, *I*^2^ values greater than 60–70% indicate the presence of substantial heterogeneity. Where substantial heterogeneity was detected, a subgroup analysis was performed to detect its possible sources using the following grouping variables: United Nations Statistical Divisions (UNSD) regions, country prevalence of HIV (≤1% versus >1%) and country level human development index. A *p* value < 0.05 was indicative of significant difference. Inter-rater agreement for study inclusion was assessed using Cohen’s κ coefficient^8^. The Egger’s test (p < 0.10) was performed to detect the presence of publication bias^9^. We synthetized factors associated with PH in people with HIV in a narrative format.

To assess the methodological quality of each study, two reviewers (J.J.B. and J.R.N.) used an adapted version of the tool developed for prevalence studies by the Joanna Briggs Institute^10^. A score of 0–3, 4–6, or 7–9 rated the risk of bias as high, moderate or low, respectively.

## Results

### The review process and study characteristics

**Figure 1:**
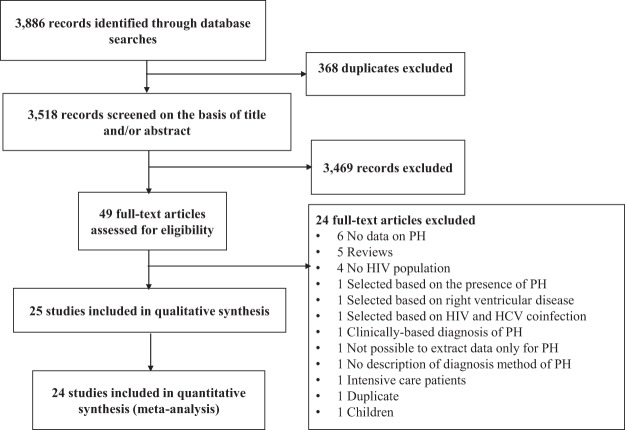
Selection of studies.

Overall, 3,886 records were initially identified. After removal of duplicates, screening of study titles, abstracts, and full texts; 25 studies were finally retained for systematic review and 24 for meta-analysis (Fig. 1)^11–35^. Concerning the methodological quality, 10 studies (40%) had a low risk of bias, 14 studies (56%) had a moderate risk and one study (4%) had a high risk of bias.

Studies were conducted in 17 countries. Participants were included from 1989 to 2015 and studies were published between 2000 and 2018. Twenty-three studies (92%) were cross-sectional and the two others (8%) were cohort studies. All studies were prospective. The mean/median age varied from 34 to 57 years for studies among adults and was 14 years for the two studies involving adolescents. The proportion of males varied from 29% to 98%. The mean/median lymphocytes T CD4 count varied from 205 to 595 cells/mm^3^ (14 studies). The proportion of undetectable HIV viral load varied from 14% to 91% (10 studies), and that of people on antiretroviral treatment, between 65% and 100% (Supplemental Table 2).

### Overall prevalence of pulmonary hypertension in the global population living with HIV

**Figure 2:**
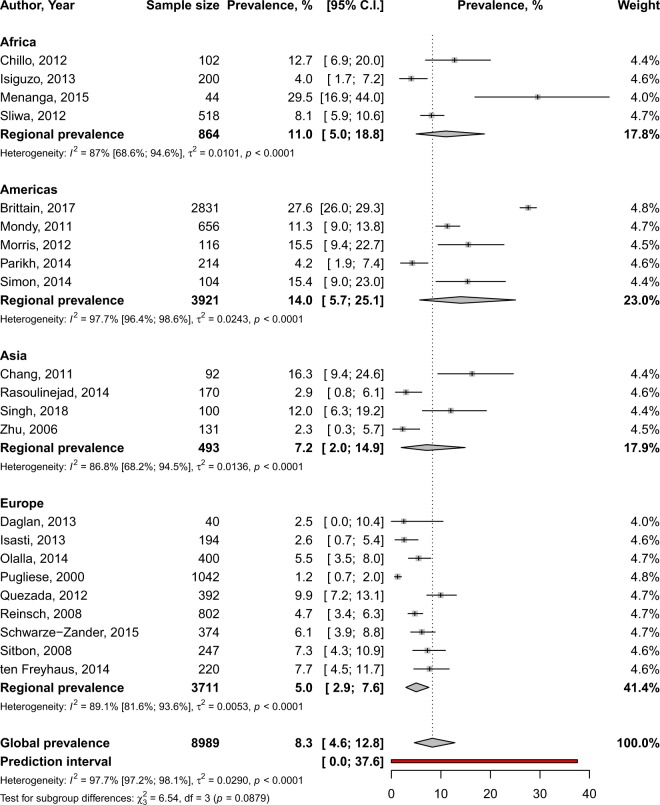
Prevalence of pulmonary hypertension in the global population of adults living with HIV by regions.

**Figure 3:**
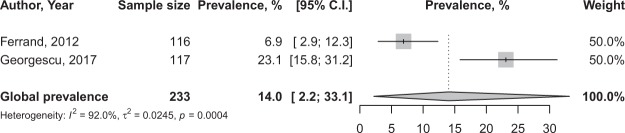
Prevalence of pulmonary hypertension in the global population of adolescents living with HIV.

In total, 42,642 participants were included. Only one study reported the prevalence of PH among HIV-infected adults based on right heart catheterization: 0.5% (95% CI: 0.3–0.6)^32^. In one study reporting data obtained based on the International Classification of Diseases, the prevalence was: 0.2% (95% CI: 0.2–0.3)^14^. The global prevalence of PH in adults with HIV, screened through transthoracic echocardiography was 8.3% (95% CI: 4.6–12.8; 22 studies; I^2^: 97.7%), varying from 1.2% in Italy to 29.5% in Cameroon with substantial heterogeneity between studies (Fig. 2). There was evidence of publication bias suggesting more publication of studies with low sample sizes which reported high prevalence estimates (Supplementary Fig. 1) confirmed by the Egger’s test, *p* = 0.061 (Table 1). The sensitive analysis including only studies with low risk of bias yielded a prevalence very close to that from crude analysis, 9.8% (95% CI: 4.4–17.0; 10 studies; I^2^: 98.1%) (Table 1). There was no difference between UNSD regions, p = 0.088 (Fig. 1), HDI category, p = 0.314 (Supplementary Fig. 2), and the burden of HIV in countries, p = 0.398 (Supplementary Fig. 3) (Table 1). Among adolescents, the prevalence of PH based on echography was 14.0% (95% CI: 2.2–33.1; I^2^: 92.0%), pooled from two studies originating from Zimbabwe and Romania (Fig. 3).

**Table 1:** Meta-analysis prevalence of echography-based pulmonary hypertension in the global population of adults living with HIV.

	Prevalence, % (95% confidence interval)	95% Prediction interval	N Studies	N Participants	H (95% confidence interval)	I^2^ (95% confidence interval)	*p* heterogeneity	*p* Egger test	*p* difference
**Global**	8.3 (4.6–12.8)	0.0–37.6	22	8989	6.6 (6.0–7.3)	97.7 (97.2–98.1)	<0.0001	0.061	—
- Low risk of bias	9.8 (4.4–17.0)	0.0–43.9	10	5910	7.4 (6.4–8.5)	98.1 (97.5–98.6)	<0.0001	0.027	—
***By regions***									
- Americas	14.0 (5.7–25.1)	0.0–64.4	5	3921	6.6 (5.2–8.3)	97.7 (96.4–98.6)	<0.0001	0.148	0.088
- Africa	11.0 (5.0–18.8)	0.0–55.3	4	864	2.8 (1.9–4.3)	87.0 (68.6–94.6)	<0.0001	0.341	
- Asia	7.2 (2.0–14.9)	0.0–56.2	4	493	2.8 (1.8–4.3)	86.8 (68.2–94.5)	<0.0001	0.100	
- Europe	5.0 (2.9–7.6)	0.0–16.0	9	3711	3.0 (2.3–4.0)	89.1 (81.6–93.6)	<0.0001	0.220	
***By human development index***									
- Low and middle	11.0 (5.9–17.4)	0.0–38.2	5	964	2.5 (1.7–3.7)	83.7 (63.0–92.8)	<0.0001	0.203	0.314
- High and very high	7.4 (4.0–12.7)	0.0–39.2	17	8025	7.5 (6.7–8.3)	98.2 (97.8–98.5)	<0.0001	0.046	
***By country HIV burden***									
- Prevalence >1%	11.0 (5.0–18.8)	0.0–55.3	4	864	2.8 (1.9–4.3)	87.0 (68.6–94.6)	<0.0001	0.341	0.398
- Prevalence ≤1%	7.6 (3.7–12.8)	0.0–39.0	18	8125	7.2 (6.5–8.0)	98.1 (97.6–98.5)	<0.0001	0.050

### Factors associated with pulmonary hypertension among people living with HIV

Independent factors associated with PH in people with HIV included female sex, detectable viral load, and HCV infection in multivariable analysis from one study^26^. Factors identified in bivariate analyses included increasing age, N-terminal-proB-type natriuretic peptide, female sex, detectable HIV viral load, hepatitis C virus infection, short duration of HIV infection, smoking, and longer exposure to antiretroviral treatment (Table 2)^12,26,28,29,34,36^.

**Table 2:** Factors associated with PH in the population with HIV infection in individual studies.

Country-Author, Year	Study characteristics	Inclusion criteria	Population	HIV	Factors searched and not identified	Factors univariate analysis	Factors multivariate analysis
Spain- Quezada, 2012	Cross sectional; Hospital-based; Prospective; Single Site; Random sampling; Inclusion 2009–2011	HIV-infected people	Age 46.9 y; 83.4% Males	Mean CD4 count 577; 84.1% ART; HIV duration 13 y; Detectable VL: 23.8%	Age; Duration of infection; Nadir CD4; Last CD4 count; HAART; HBV	Female; IDU; Detectable HIV RNA; HCV	Female; Detectable HIV VL; HCV
Germany - ten Freyhaus, 2014	Cross sectional; Hospital-based; Prospective; Single Site; Consecutive sampling	HIV-infected people routinely followed	Age 44.0 y; 84.1% Males; HCV: 1%; HBV; 0.5%	Mean CD4 count 400; VL <400 copies/mL: 75%	Age; HIV stage disease; HIV viral load; CD4 count; CD4/CD8	High NT-proBNP	
Germany - Schwarze-Zander, 2015	Cross sectional; Hospital-based; Prospective; Single Site; Consecutive sampling; Inclusion 2009–2012	HIV-infected people	Age 46.0 y; 80% Males; IDU: 7%	Mean CD4 count 476; 90% ART; HIV duration 8.4 y; VL <40 copies/mL: 75%	Age; Sex; HIV stage; ART; CD4	Low duration HIV; High NT-proBNP	
India – Singh, 2018	Cross sectional; Hospital-based; Prospective; Single Site; Consecutive sampling; Inclusion 2014–2015	>18 years. Excluded: chronic heart failure, diabetes mellitus, hypothyroidism, patient on steroid, history of myocardial infarction, congenital heart diseases	Age 34.3 y; 55% Males	CD4 count <350: 46%;; HIV duration 1.8 y	CD4 count		
South Korea – Chang, 2011	Cross sectional; Hospital-based; Prospective; Single Site; Consecutive sampling; Inclusion 2010	Asymptomatic HIV	Age 45.3 y; 95.7% Males	CD4 count 495; 84.8% ART; HIV duration 5.6 y	Sex, Age, AIDS on presentation, CD4 count, HIV viral load, HIV duration, HAART		
Spain - Isasti, 2013	Cross sectional; Hospital-based; Prospective; Single Site; Consecutive sampling; Inclusion 2011	≥18 years. Exclusion: prior known structural heart disease of any aetiology; pregnant or lactating women.	Age 49.0 y; 88% Males	CD4 count 550; 97% ART; HIV duration 12.5 y; VL <50 copies/ml: 90%	Sex; Alcohol; HCV; Diabetes; Prior pulmonary disease; AIDS event; Time with HIV; HAART; HIV viral load; CD4 count	Increased Age, Smoking, Increased time on ART	
Europe – Reinsch, 2008	Cross sectional; Hospital-based; Prospective; Multisite; Consecutive sampling	Excluded: inability or unwillingness to give informed consent to participate in the study, <18 years, an unstable cardiovascular status in the 4 weeks prior to the screening visit, current hospitalization and pregnancy.	Age 44.0 y; 83.4% Males	CD4 count 508; 85.3% ART;; HIV duration 7.75 y	Sex; HCV; HBV; Race; Duration of HIV; ART; HIV Viral Load; CD4 count	Increased age	

AIDS: acquired immunodeficiency virus; ART: antiretroviral treatment; CD4: lymphocytes T CD4; HAART: highly active antiretroviral treatment; HBV: hepatitis B virus infection; HCV: hepatitis C virus infection; IDU: injecting drug use; VL: viral load.

## Discussion

This first global systematic review and meta-analysis of data from 42,642 adolescents and adults living with HIV from 17 countries showed an overall PH prevalence of 8.3% in adults and 14.0% in adolescents (based on transthoracic echocardiography), with substantial heterogeneity between studies. Only one study reported a PH prevalence of 0.5% among HIV-infected adults; the diagnosis was based on right heart catheterisation. There was no difference according to human development index, UNSD regions, and HIV burden in countries for echography-based PH.

In the general global population, the prevalence of PH is around 1%^2^. The updated comprehensive clinical classification of PH by Simonneau and colleagues includes in group 1, pulmonary arterial hypertension associated with HIV as one type of PH^37^. In this study, we found a higher prevalence of PH in the global population living with HIV compared to the general global population. Evidence suggests HIV infection as the leading infectious cause of PH^2^. Worldwide, there are 36.9 millions of people living with HIV suggesting that there may be more than 3 millions of people living with HIV experiencing PH, considering our findings for echography-based PH. However, there is a disparity in the burden of HIV worldwide and this should be taken into account. Indeed, in the top 25 countries with the highest burden of HIV infection, there are 23 countries from sub-Saharan Africa where HIV prevalence exceeds 20% in some areas^38^. Although without significant difference, we found higher prevalence of PH in people living with HIV in Africa and Americas; however, this finding should be interpreted with caution since few studies were reported from each region and no study was carried-out in Oceania.

As suggested by animal model studies, basic science studies in humans, and well-established classification of aetiologies of PH, exposure to HIV infection is considered as an aetiology of PH^2,39^. To date, evidence suggests a complex interplay between direct HIV infection in the lung and chronic inflammation as pathophysiological elements to explain the development of PH in people with HIV^39^. Macrophages circulating in the lung serve as reservoirs for the transmission of HIV to circulating T-lymphocytes favouring the presence of HIV viral proteins in lungs (Nef, Tat, gp120)^39^. As we found in this review, detectable viral load favoured the occurrence of PH in people with HIV but this was not the case for lymphocytes T CD4 counts. These HIV-proteins increased the susceptibility to develop vascular oxidative stress, apoptosis, smooth myocyte proliferation, and endothelial cells injury. Gp120 significantly increases secretion of the potent vasoconstrictor endothelin-1 by human lung endothelial cells^40^. HIV infection itself induces high and chronic secretion of some cytokines (interleukins (IL)-1, IL-6, IL8, IL-13, tumour necrosis factor-α and platelet-derived growth factors) which can induce chronic inflammation of vascular endothelium leading to PH^40^. The higher prevalence of PH in people living with HIV may also be associated with the higher proportion of other factors associated with PH including injecting drug use, HCV infection, and smoking habits.

Our findings have important implications for healthcare providers, health policy makers and researchers. Policy makers should be aware of the high prevalence of PH in people living with HIV in order to better prepare and strengthen health care systems for a proper management of this condition in this vulnerable population which may undergo pharmacological interactions of drugs for PH and ART. With more than 70% of people living with HIV in sub-Saharan Africa, this region deserves special attention from policy makers and healthcare workers since one may find more than 2 million people living with both HIV and PH. Before integrating PH screening in routine care in HIV clinics in Africa where healthcare systems are (very) weak and resources are limited, well-designed cost-effective studies are needed. These studies would contribute to bring evidence with the aim of helping to achieve the Sustainable Development Goal 3: “Ensure healthy lives and promote well-being for all at all ages”^41^. We identified one study with multivariable model that identified detectable HIV viral load as a factor associated with PH in people living with HIV. It is well known that antiretroviral treatment reduces HIV viral load. Majority of studies demonstrated the beneficial effect of using highly active antiretroviral therapy (HAART) among HIV infected patients with PH on their outcome, especially if initiated at an early stage of HIV infection^42^. Since it is difficult or impossible to implement a systematic screening of PH using the right heart catheterisation because this is an invasive method, it is important to set up a diagnostic algorithm to identify as soon as possible all HIV people who may have PH, at an early stage.

Notwithstanding, this study should be interpreted in the context of some drawbacks. First, most studies used only echography to identify people with PH. This may have led to an overestimation of the PH prevalence. However, this is the less invasive diagnosis method and less expensive compared to right heart catheterisation. Second, only 10 of 23 included studies had low risk of bias; however, our analysis including only studies with low risk of bias yielded an estimate closer to that from the crude prevalence. Third, the various geographic regions and countries were variably represented, which could have affected the generalizability of our findings. Despite these limitations, this is to the very best of our knowledge the first systematic review and meta-analysis providing a global estimate of the burden of PH in people living with HIV. We used rigorous methodological and statistical procedures to obtain and pool our data. Furthermore, subgroup analyses were conducted to investigate the various factors likely affecting our estimates.

This systematic review and meta-analysis compiled data from 17 countries and pointed a high prevalence of PH in people living with HIV. As such, PH in people living with HIV should be prioritized among HIV health care providers, policy makers and stakeholders from the health sector for improved detection, overall proper management and efficient control. This estimate seemed not different with regard to UNSD regions, country human development index, and HIV burden in the country. Studies are needed to investigate cost-effective strategies to curb the burden of PH in people living in HIV, especially in Africa where more than 70% of HIV-infected people reside and where most countries have weak health care systems. Moreover, co-located non-communicable disease services for individuals enrolled in HIV care and treatment should be investigated for PH.

## Supplementary information

Appendix

## Data Availability

All data relevant to the study are included in the article or uploaded as Supplementary Information.

## References

[1] Hoeper MM (2013). Definitions and diagnosis of pulmonary hypertension. Journal of the American College of Cardiology.

[2] Hoeper MM (2016). A global view of pulmonary hypertension. Lancet Respir Med.

[3] UNAIDS. Chronic care of HIV and noncommunicable diseases: How to leverage the HIV experience, http://www.unaids.org/sites/default/files/media_asset/20110526_JC2145_Chronic_care_of_HIV_0.pdf (2011).

[4] Degano B (2010). HIV-associated pulmonary arterial hypertension: survival and prognostic factors in the modern therapeutic era. AIDS (London, England).

[5] Moher D, Liberati A, Tetzlaff J, Altman DG (2009). Preferred reporting items for systematic reviews and meta-analyses: the PRISMA statement. BMJ (Clinical research ed.).

[6] Barendregt JJ, Doi SA, Lee YY, Norman RE, Vos T (2013). Meta-analysis of prevalence. Journal of epidemiology and community health.

[7] Higgins JP, Thompson SG, Deeks JJ, Altman DG (2003). Measuring inconsistency in meta-analyses. BMJ (Clinical research ed.).

[8] Viera AJ, Garrett JM (2005). Understanding interobserver agreement: the kappa statistic. Family medicine.

[9] Egger M, Davey Smith G, Schneider M, Minder C (1997). Bias in meta-analysis detected by a simple, graphical test. BMJ (Clinical research ed.).

[10] The Joanna Briggs Institute. The Joanna Briggs Institute Critical Appraisal tools for use in JBI Systematic Reviews: Checklist for Prevalence Studies, http://joannabriggs.org/research/critical-appraisal-tools.html (2017).

[11] Brittain EL (2018). Increased Echocardiographic Pulmonary Pressure in HIV-infected and -uninfected Individuals in the Veterans Aging Cohort Study. American journal of respiratory and critical care medicine.

[12] Chang H-J (2011). Prevalence and Clinical Characteristics of Pulmonary Arterial Hypertension in Human Immunodeficiency Virus-Infected Patients. The Korean Journal of Medicine.

[13] Chillo P, Bakari M, Lwakatare J (2012). Echocardiographic diagnoses in HIV-infected patients presenting with cardiac symptoms at Muhimbili National Hospital in Dar es Salaam, Tanzania. Cardiovascular journal of Africa.

[14] Crothers K (2011). HIV infection and risk for incident pulmonary diseases in the combination antiretroviral therapy era. American journal of respiratory and critical care medicine.

[15] Daglan E, Yamin D, Manu B, Streinu-Cercel A (2013). Cardiac involvement in HIV-positive patients. Germs.

[16] Ferrand RA (2012). Chronic lung disease in adolescents with delayed diagnosis of vertically acquired HIV infection. Clinical infectious diseases: an official publication of the Infectious Diseases Society of America.

[17] Georgescu AM, Moldovan C, Szederjesi J, Georgescu D, Azamfirei L (2017). Echocardiographic characteristics of pulmonary arterial hypertension in children with horizontally transmitted HIV. Advances in clinical and experimental medicine: official organ Wroclaw Medical University.

[18] Isasti G (2013). Echocardiographic abnormalities and associated factors in a cohort of asymptomatic HIV-infected patients. AIDS research and human retroviruses.

[19] Isiguzo GC (2013). Contributions of pulmonary hypertension to HIV-related cardiac dysfunction. Indian heart journal.

[20] Menanga AP (2015). Patterns of cardiovascular disease in a group of HIV-infected adults in Yaounde, Cameroon. Cardiovascular diagnosis and therapy.

[21] Mondy KE (2011). High Prevalence of Echocardiographic Abnormalities among HIV-infected Persons in the Era of Highly Active Antiretroviral Therapy. Clinical infectious diseases: an official publication of the Infectious Diseases Society of America.

[22] Morris A (2012). Cardiopulmonary function in individuals with HIV infection in the antiretroviral therapy era. AIDS (London, England).

[23] Olalla J (2014). Pulmonary hypertension in human immunodeficiency virus-infected patients: the role of antiretroviral therapy. Medicina clinica.

[24] Parikh RV (2014). Increased levels of asymmetric dimethylarginine are associated with pulmonary arterial hypertension in HIV infection. AIDS (London, England).

[25] Pugliese A (2000). Impact of highly active antiretroviral therapy in HIV-positive patients with cardiac involvement. The Journal of infection.

[26] Quezada M (2012). Prevalence and risk factors associated with pulmonary hypertension in HIV-infected patients on regular follow-up. AIDS (London, England).

[27] Rasoulinejad M (2014). Echocardiographic assessment of systolic pulmonary arterial pressure in HIV-positive patients. Acta medica Iranica.

[28] Reinsch N (2008). Effect of gender and highly active antiretroviral therapy on HIV-related pulmonary arterial hypertension: results of the HIV-HEART Study. HIV medicine.

[29] Schwarze-Zander C (2015). Pulmonary hypertension in HIV infection: a prospective echocardiographic study. HIV medicine.

[30] Simon MA (2014). Isolated right ventricular dysfunction in patients with human immunodeficiency virus. Journal of cardiac failure.

[31] Singh S, Vatsa D, Tomar S, Aneja GK, Tv SA (2018). Cardiac complications in people living with human immunodeficiency virus/acquired immunodeficiency syndrome and their association with CD4+ T-cell count - A cross sectional study. Indian journal of sexually transmitted diseases and AIDS.

[32] Sitbon O (2008). Prevalence of HIV-related pulmonary arterial hypertension in the current antiretroviral therapy era. Am J Respir Crit Care Med.

[33] Sliwa K (2012). Contribution of the human immunodeficiency virus/acquired immunodeficiency syndrome epidemic to *de novo* presentations of heart disease in the Heart of Soweto Study cohort. European heart journal.

[34] ten Freyhaus H (2014). Echocardiographic screening for pulmonary arterial hypertension in HIV-positive patients. Infection.

[35] Zhu HH (2006). Echocardiographic study of HIV positive/AIDS patients. *Chinese*. Journal of Medical Imaging Technology.

[36] Isasti G (2013). High prevalence of pulmonary arterial hypertension in a cohort of asymptomatic HIV-infected patients. AIDS research and human retroviruses.

[37] Galie N (2015). ESC/ERS Guidelines for the diagnosis and treatment of pulmonary hypertension: The Joint Task Force for the Diagnosis and Treatment of Pulmonary Hypertension of the European Society of Cardiology (ESC) and the European Respiratory Society (ERS): Endorsed by: Association for European Paediatric and Congenital Cardiology (AEPC), International Society for Heart and Lung Transplantation (ISHLT). The European respiratory journal.

[38] UNAIDS. Global HIV Statistics, http://www.unaids.org/sites/default/files/media_asset/UNAIDS_FactSheet_en.pdf (2018).

[39] Hassoun PM (2009). Inflammation, growth factors, and pulmonary vascular remodeling. Journal of the American College of Cardiology.

[40] Bigna JJ, Sime PS, Koulla-Shiro S (2015). HIV related pulmonary arterial hypertension: epidemiology in Africa, physiopathology, and role of antiretroviral treatment. AIDS Res Ther.

[41] United Nations. Sustained Development Goals 3: Ensure healthy lives and promote well-being for all at all ages, https://www.un.org/sustainabledevelopment/health/.

[42] Pal J (2013). Effect of antiretroviral therapy on pulmonary hypertension in HIV patients. Journal of the Indian Medical Association.

