# Supplementary material for: Pulmonary hypertension in the global population of adolescents and adults living with HIV: a systematic review and meta-analysis
Source: Sci Rep. 2019 May 24;9:7837. doi: 10.1038/s41598-019-44300-5 (PMC6534533; doi:10.1038/s41598-019-44300-5)

Pulmonary hypertension in the global population of adolescents and adults living with HIV: a systematic review and meta-analysis

Jean Joel Bigna, MD, MPH; Jobert Richie Nansseu, MD; Jean Jacques Noubiap, MD

APPENDIX

Supplementary Table 1. Search strategy in PubMed 2
Supplementary Table 2. Individual characteristics of included studies 3

Supplementary Figure 1. Funnel plot for publication bias 6
Supplementary Figure 2. Meta-analysis prevalence of pulmonary hypertension in adults living with HIV by human development index 7
Supplementary Figure 3. Meta-analysis prevalence of pulmonary hypertension in adults living with HIV by burden of HIV in countries 8

Supplementary Table 1. Search strategy in PubMed

Search	Search terms
#1	pulmonary hypertension OR pulmonary arterial hypertension
#2	HIV OR AIDS OR human immunodeficiency virus OR acquired immunodeficiency virus
#3	#1 AND #2

Supplementary Table 2. Individual characteristics of included studies

Author	Year of publication	Study Design	Period of inclusion	Country	#Centers	Site	Sampling	Timing	Mean /Median age, years	%Males	CD4 count	Viral Load	Time HIV in years	%ART	Other characteristics	PH Diagnostic method	Sample	Risk of bias
**Brittain**	2017	Cohort	2003	USA	Multi-Site study	Hospital-based	Unclear	Prospectively	57	97	CD4<200:22%; 200<CD4<500:44%; CD4>500: 33%	VL<500:65%; 500<VL<1000: 3%; VL>1000: 32%	NR	65		Doppler echocardiography	2831	Low
**Chang**	2011	Cross sectional	2010	South Korea	Single-Site study	Hospital-based	Consecutive	Prospectively	45.4	95.7	495	3772	5.6	84.8		Doppler echocardiography	92	Low
**Chillo**	2012	Cross sectional	2009-2010	Tanzania	Single-Site study	Hospital-based	Consecutive	Prospectively	42	31.4	CD4<200: 25%	NR	Time > 1 year: 60.8%	68.6	Smoking: 8.8%; Alcohol: 25.5%; Diabetes: 5.9%	Doppler echocardiography	102	Low
**Crothers**	2011	Cross sectional	2007	USA	Multi-Site study	Population-based	Unclear	Prospectively	45	98	264	14815; VL<400: 14%	NR	65		ICD-9	33420	Moderate
**Daglan**	2013	Cross sectional	2011-2012	Romania	Single-Site study	Hospital-based	Random	Prospectively	Age>30 ans:27.5%	42.5	NR	NR	Duration >21 years: 77.5%	100	Smoking Yes (10 pack-years): 60%; Alcohol use: 22.5	Doppler echocardiography	40	Low
**Ferrand**	2012	Cross sectional	NR	Zimbabwe	Multi-Site study	Hospital-based	Consecutive	Prospectively	14	43	384	NR	NR	69		Doppler echocardiography	116	Moderate
**Georgescu**	2017	Cohort	NR	Romania	Single-Site study	Hospital-based	Consecutive	Prospectively	14	49.6	NR	NR	NR	NR		Doppler echocardiography	117	Moderate
**Isasti**	2013	Cross sectional	2011	Spain	Single-Site study	Hospital-based	Consecutive	Prospectively	49	88	550	VL < 50 copies: 90%	125	97		Doppler echocardiography	194	Low
**Isiguzo**	2013	Cross sectional	2010	Nigeria	Single-Site study	Hospital-based	Consecutive	Prospectively	39	29	NR	NR	NR	NR	Alcohol: 23%	Doppler echocardiography	200	Low
**Menanga**	2015	Cross sectional	2014	Cameroon	Multi-Site study	Hospital-based	Consecutive	Prospectively	48	48	205	NR	Duration>1 year: 54.5%	70.5	WHO clinical stage. WHO1: 13.6%; WHO2: 18.2%; WHO3: 25.0%; WHO4: 70.5%	Doppler echocardiography	44	Low

Author	Year of publication	Study Design	Period of inclusion	Country	#Centers	Site	Sampling	Timing	Mean /Median age, years	%Males	CD4 count	Viral Load	Time HIV in years	%ART	Other characteristics	PH Diagnostic method	Sample	Risk of bias
**Mondy**	2011	Cross sectional	2004-2006	USA	Multi-Site study	Hospital-based	Consecutive	Prospectively	41	76	462	VL< 400: 91%	NR	91		Doppler echocardiography	656	Low
**Morris**	2012	Cross sectional	2007-2010	USA	Multi-Site study	Hospital-based	Consecutive	Prospectively	47.7	69.8	578	VL < 50: 27.6%	NR	88.8	Hepatitis: 13.0%; Ever smoker: 81.9%; IDU in the past 6 months: 0.9%	Doppler echocardiography	116	Low
**Olalla**	2014	Cross sectional	2009-2011	Spain	Single-Site study	Hospital-based	Consecutive	Prospectively	45.6	77	595	NR	NR	100	HBV: 18.5%; HCV: 32.5%	Doppler echocardiography	400	Low
**Parikh**	2014	Cross sectional	NR	USA	Single-Site study	Unclear	Consecutive	Prospectively	50	82	586	VL< 15 copies/L: 71%	15		IVDU: 8%; Diabetes 11%; Ever smokers: 65%; Current smokers: 36%; HCV: 25%;	Doppler echocardiography	214	Moderate
**Pugliese**	2000	Cross sectional	1989-1998	Italy	Single-Site study	Hospital-based	Consecutive	Prospectively	35.5	77.3	NR	NR	NR	100		Doppler echocardiography	1042	Low
**Quezada**	2012	Cross sectional	2009-2011	Spain	Single-Site study	Hospital-based	Random	Prospectively	46.9	83.4	577	Detectable VL: 23.8	13	84.1		Doppler echocardiography	392	Low
**Rasoulinejad**	2014	Cross sectional	2011-2013	Iran	Single-Site study	Hospital-based	Consecutive	Prospectively	41	63.5	401	NR	5.5	100	Smokers: 48%; IDU: 50.5%; Diabetes: 4.5%; HCV: 48%	Doppler echocardiography	170	Moderate
**Reinsch**	2008	Cross sectional	NR	Middle Europe	Multi-Site study	Hospital-based	Consecutive	Prospectively	44	83.4	508	34973	7.75	85.3		Doppler echocardiography	802	Low
**Schwarze-Zander**	2015	Cross sectional	2009-2012	Germany	Single-Site study	Hospital-based	Consecutive	Prospectively	46	80	476	VL<40: 75%	8.4	90	IDU: 7%	Doppler echocardiography	374	Low
**Simon**	2014	Cross sectional	2009-2013	USA	Multi-Site study	Hospital-based	Consecutive	Prospectively	47	71	591	Log VL: 2.1	NR	89	Smoking history: 81%; Drug use: 3%;	Doppler echocardiography	104	Low
**Singh**	2018	Cross sectional	2014-2015	India	Single-Site study	Hospital-based	Consecutive	Prospectively	34.3	55	CD4 count < 350: 46%	NR	1.8	NR		Doppler echocardiography	100	Low

Author	Year of publication	Study Design	Period of inclusion	Country	#Centers	Site	Sampling	Timing	Mean /Median age, years	%Males	CD4 count	Viral Load	Time HIV in years	%ART	Other characteristics	PH Diagnostic method	Sample	Risk of bias
**Sitbon**	2008	Cross sectional	2004-2005	France	Multi-Site study	Hospital-based	Unclear	Prospectively	42.3	55	CD4 < 200: 20%	VL< 400: 66%	10	86		Doppler echocardiography	277	Low
**Sliwa**	2012	Cross sectional	2006-2008	South Africa	Multi-Site study	Hospital-based, Population-based	Consecutive	Prospectively	40	38	NR	NR	NR	NR		Doppler echocardiography	518	Low
**ten Freyhaus**	2014	Cross sectional	NR	Germany	Single-Site study	Hospital-based	Consecutive	Prospectively	44	84.1	400	VL < 400: 75%	NR	NR	HCV: 1%; HBV; 0.5	Doppler echocardiography	220	Low
**Zhu**	2006	Cross sectional	NR	China	Single-Site study	Hospital-based	Unclear	Prospectively	NR	NR	NR	NR	NR	NR		Doppler echocardiography	131	High

Supplementary Figure 1. Funnel plot for publication bias

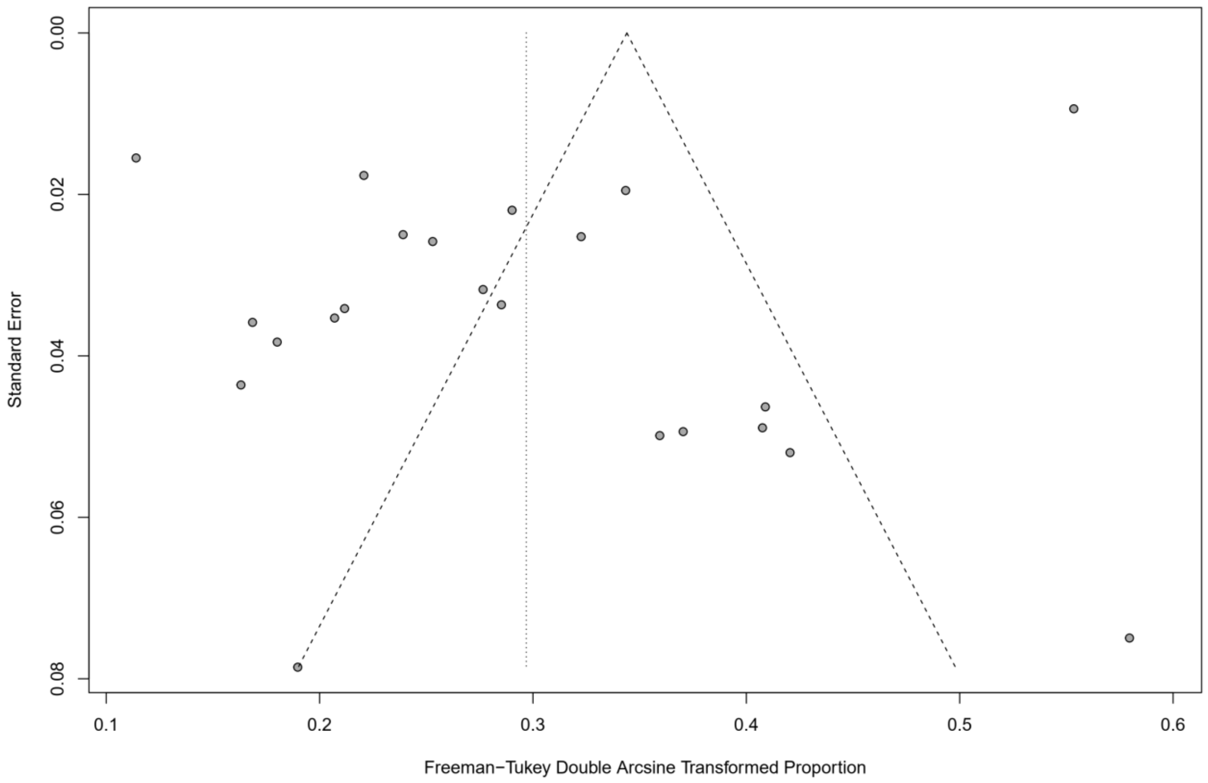

Studies are missing in the right-top side of the figure suggesting the presence of publication bias with more publication of studies with low sample size which reported high prevalence estimates

Supplementary Figure 2. Meta-analysis prevalence of pulmonary hypertension in adults living with HIV by human development index

Author, Year	Sample size	Prevalence, %	[95% C.I.]	Weight
**High and Very high**				
Brittain, 2017	2831	27.6	[26.0; 29.3]	4.8%
Chang, 2011	92	16.3	[9.4; 24.6]	4.4%
Daglan, 2013	40	2.5	[0.0; 10.4]	4.0%
Isasti, 2013	194	2.6	[0.7; 5.4]	4.6%
Mondy, 2011	656	11.3	[9.0; 13.8]	4.7%
Morris, 2012	116	15.5	[9.4; 22.7]	4.5%
Olalla, 2014	400	5.5	[3.5; 8.0]	4.7%
Parikh, 2014	214	4.2	[1.9; 7.4]	4.6%
Pugliese, 2000	1042	1.2	[0.7; 2.0]	4.8%
Quezada, 2012	392	9.9	[7.2; 13.1]	4.7%
Rasoulinejad, 2014	170	2.9	[0.8; 6.1]	4.6%
Reinsch, 2008	802	4.7	[3.4; 6.3]	4.7%
Schwarze−Zander, 2015	374	6.1	[3.9; 8.8]	4.7%
Simon, 2014	104	15.4	[9.0; 23.0]	4.4%
Sitbon, 2008	247	7.3	[4.3; 10.9]	4.6%
ten Freyhaus, 2014	220	7.7	[4.5; 11.7]	4.6%
Zhu, 2006	131	2.3	[0.3; 5.7]	4.5%
**Regional prevalence**	**8025**	**7.4**	**[3.4; 12.7]**	**77.8%**
Heterogeneity: I^2 = 98.2% [97.8%; 98.5%], τ^2 = 0.0327, p < 0.0001				
**Low and Middle**				
Chillo, 2012	102	12.7	[6.9; 20.0]	4.4%
Isiguzo, 2013	200	4.0	[1.7; 7.2]	4.6%
Menanga, 2015	44	29.5	[16.9; 44.0]	4.0%
Singh, 2018	100	12.0	[6.3; 19.2]	4.4%
Sliwa, 2012	518	8.1	[5.9; 10.6]	4.7%
**Regional prevalence**	**964**	**11.0**	**[5.9; 17.4]**	**22.2%**
Heterogeneity: I^2 = 83.7% [63%; 92.8%], τ^2 = 0.0082, p < 0.0001				
**Global prevalence**	**8989**	**8.3**	**[4.6; 12.8]**	**100.0%**
**Prediction interval**			**[0.0; 37.6]**	
Heterogeneity: I^2 = 97.7% [97.2%; 98.1%], τ^2 = 0.0290, p < 0.0001				
Test for subgroup differences: χ^2_1 = 1.02, df = 1 (p = 0.3137)				

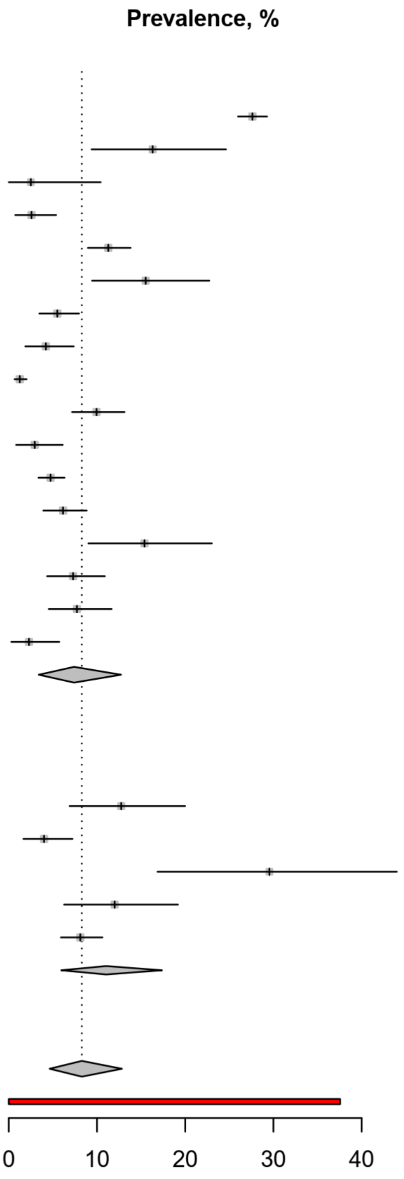

Supplementary Figure 3. Meta-analysis prevalence of pulmonary hypertension in adults living with HIV by burden of HIV in countries

Author, Year	Sample size	Prevalence, %	[95% C.I.]	Weight
**Country HIV prevalence < 1%**				
Brittain, 2017	2831	27.6	[26.0; 29.3]	4.8%
Chang, 2011	92	16.3	[9.4; 24.6]	4.4%
Daglan, 2013	40	2.5	[0.0; 10.4]	4.0%
Isasti, 2013	194	2.6	[0.7; 5.4]	4.6%
Mondy, 2011	656	11.3	[9.0; 13.8]	4.7%
Morris, 2012	116	15.5	[9.4; 22.7]	4.5%
Olalla, 2014	400	5.5	[3.5; 8.0]	4.7%
Parikh, 2014	214	4.2	[1.9; 7.4]	4.6%
Pugliese, 2000	1042	1.2	[0.7; 2.0]	4.8%
Quezada, 2012	392	9.9	[7.2; 13.1]	4.7%
Rasoulinejad, 2014	170	2.9	[0.8; 6.1]	4.6%
Reinsch, 2008	802	4.7	[3.4; 6.3]	4.7%
Schwarze−Zander, 2015	374	6.1	[3.9; 8.8]	4.7%
Simon, 2014	104	15.4	[9.0; 23.0]	4.4%
Singh, 2018	100	12.0	[6.3; 19.2]	4.4%
Sitbon, 2008	247	7.3	[4.3; 10.9]	4.6%
ten Freyhaus, 2014	220	7.7	[4.5; 11.7]	4.6%
Zhu, 2006	131	2.3	[0.3; 5.7]	4.5%
**Regional prevalence**	**8125**	**7.6**	**[3.7; 12.8]**	**82.2%**
Heterogeneity: I^2 = 98.1% [97.6%; 98.5%], τ^2 = 0.0321, p < 0.0001				
**Country HIV prevalence >=1%**				
Chillo, 2012	102	12.7	[6.9; 20.0]	4.4%
Isiguzo, 2013	200	4.0	[1.7; 7.2]	4.6%
Menanga, 2015	44	29.5	[16.9; 44.0]	4.0%
Sliwa, 2012	518	8.1	[5.9; 10.6]	4.7%
**Regional prevalence**	**864**	**11.0**	**[5.0; 18.8]**	**17.8%**
Heterogeneity: I^2 = 87% [68.6%; 94.6%], τ^2 = 0.0101, p < 0.0001				
**Global prevalence**	**8989**	**8.3**	**[4.6; 12.8]**	**100.0%**
**Prediction interval**			**[0.0; 37.6]**	
Heterogeneity: I^2 = 97.7% [97.2%; 98.1%], τ^2 = 0.0290, p < 0.0001				
Test for subgroup differences: χ^2_1 = 0.71, df = 1 (p = 0.3981)				

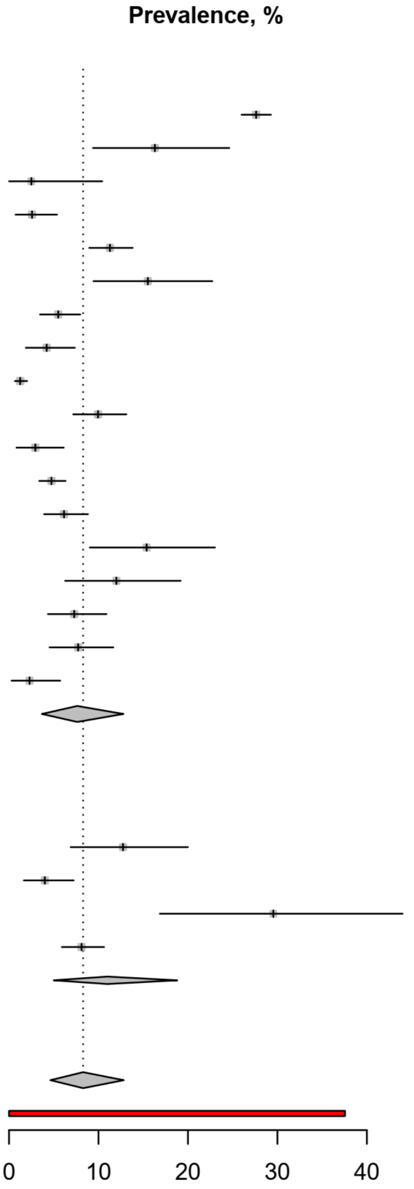

Supplement: Supplementary file 1 — Appendix [file 41598_2019_44300_MOESM1_ESM.pdf]
